# UHPLC-MS/MS Analysis of Cannabidiol and Its Metabolites in Serum of Patients with Resistant Epilepsy Treated with CBD Formulations

**DOI:** 10.3390/ph14070630

**Published:** 2021-06-29

**Authors:** Sara Malaca, Massimo Gottardi, Federica Pigliasco, Sebastiano Barco, Alessia Cafaro, Elisabetta Amadori, Antonella Riva, Martina Marcenaro, Pasquale Striano, Giuliana Cangemi, Roberta Pacifici, Simona Pichini, Francesco Paolo Busardò

**Affiliations:** 1 Department of Excellence-Biomedical Sciences and Public Health, Università Politecnica delle Marche, 60121 Ancona, Italy; smalaca@hotmail.com (S.M.); fra.busardo@libero.it (F.P.B.); 2 Comedical S.Rl, 38123 Trento, Italy; massimo.gottardi@comedical.biz; 3 Chromatography and Mass Spectrometry Section, Central Laboratory of Analyses, IRCCS Istituto Giannina Gaslini, 16147 Genoa, Italy; federicapigliasco@gaslini.org (F.P.); sebastianobarco@gaslini.org (S.B.); alessiacafaro@gaslini.org (A.C.); 4 Pediatric Neurology and Muscolar Diseases Unit, IRCCS Istituto Giannina Gaslini, 16147 Genoa, Italy; elyamadori@libero.it (E.A.); riva.anto94@gmail.com (A.R.); pasqualestriano@gaslini.org (P.S.); 5 Department of Neurosciences, Rehabilitation, Ophtalmology, Genetics, Maternal and Child Health, University of Genoa, 16126 Genoa, Italy; marcenaro.martina@gmail.com; 6 National Centre on Addiction and Doping, Istituto Superiore di Sanità, 00161 Rome, Italy; roberta.pacifici@iss.it (R.P.); simona.pichini@iss.it (S.P.)

**Keywords:** cannabinoids, medical cannabis, serum, CBD metabolites, UHPLC-MS/MS

## Abstract

Cannabidiol (CBD) is a promising therapeutic agent with analgesic, myorelaxant, and anti-epileptic actions. Recently, a purified form of CBD (Epidiolex^®^) has been approved by the European Medicines Agency (EMA) for the treatment of two highly-refractory childhood-onset epilepsies (Dravet and Lennox-Gastaut syndrome). Given the interindividual response and the relationship between the dose administered and CBD blood levels, therapeutic drug monitoring (TDM) is a valuable support in the clinical management of patients. We herein report for the first time a newly developed and validated method using ultra-high-performance liquid chromatography coupled with tandem mass spectrometry (UHPLC–MS/MS) to evaluate CBD and its metabolites (i.e., cannabidiol-7-oic acid (7-COOH-CBD), 7-hydroxycannabidiol (7-OH-CBD), 6-α-hydroxycannabidiol (6-α–OH–CBD) and 6-β-hydroxycannabidiol (6-β–OH–CBD)) in serum samples. The method reached the sensitivity needed to detect minimal amounts of analytes under investigation with limits of quantification ranging from 0.5 to 20 ng/mL. The validation results indicated in this method were accurate (average inter/intra-day error, <15%), precise (inter/intra-day imprecision, <15%), and fast (8 min run time). The method resulted to be linear in the range of 1–10,000 ng/mL for CBD-COOH, 1–500 ng/mL for 7-OH-CBD and CBD and 1–25 ng/mL for 6-α–OH–CBD and 6-β–OH–CBD. Serum levels of CBD (88.20–396.31 and 13.19–170.63 ng/mL) as well as of 7-OH-CBD (27.11–313.63 and 14.01–77.52 ng/mL) and 7-COOH-CBD (380.32–10,112.23 and 300.57–2851.82 ng/mL) were significantly higher (*p* < 0.05) in patients treated with GW pharma CBD compared to those of patients treated with galenic preparations. 6-α–OH–CBD and 6-β–OH–CBD were detected in the first group and were undetectable in the second group. 7-COOH-CBD was confirmed as the most abundant metabolite in serum (5–10 fold higher than CBD) followed by 7-OH-CBD. A significant correlation (*p* < 0.05) between the dose administrated and a higher bioavailability was confirmed in patients treated with a GW pharma CBD preparation.

## 1. Introduction

Δ^9^-tetrahydrocannabinol (THC) and cannabidiol (CBD) are the most investigated phytocannabinoids due to their pharmacological activity [1,2] even if they display different proprieties. Multiple possible pharmacological targets exist for CBD, but few have been verified. Additionally, CBD has shown antiepileptic, anti-inflammatory, anxiolytic, and neuroprotective proprieties without psychotropic or addictive effects than those expected from THC [3,4,5,6,7,8,9] Although recreational cannabis mainly contains THC, medical cannabis can contain both THC and CBD at different ratios, together with minor phytocannabinoids and terpenes [9]. The combination of both cannabinoids with the other constituents of the phytocomplex is most likely the reason for the efficacy of cannabis-based medicinal extracts and the lower occurrence of side effects if compared with synthetic cannabinoids [10,11]. All these components contribute to the different pharmacological effects of medical cannabis fund either the treatment of neuropathic pain, cancer, insomnia, and epilepsy [3]. CBD oil oral solution, commercialized as Epidiolex^®^, is currently used for the treatment of two rare and severe forms of epilepsy, namely Lennox-Gastaut syndrome (LGS) [12,13] and Dravet syndrome (DS) [14,15]. Epidiolex^®^ was the first U.S. Food and Drug Administration (FDA)-approved drug, containing a purified drug substance derived from cannabis, was scheduled as a “drug with lower potential for abuse than Schedule IV containing limited quantities of certain narcotics” [16].

Previous studies have determined the levels of CBD or its metabolites in biological samples after medical cannabis consumption [17,18,19,20,21,22]. Although these studies were valuable, no analytical assay exists for the simultaneous determination of CBD and its main metabolites, 7-hydroxycannabidiol (7-OH-CBD), 6-α-hydroxycannabidiol (6-α–OH–CBD) and 6-β-hydroxycannabidiol (6-β–OH–CBD) in serum samples of individuals treated with CBD-based pharmaceuticals or medical cannabis. We present a validated UHPLC-MS/MS method to determine the above-mentioned compounds and their application to real serum samples from patients treated for refractory epilepsy.

## 2. Results and Discussion

**Figure 1:**
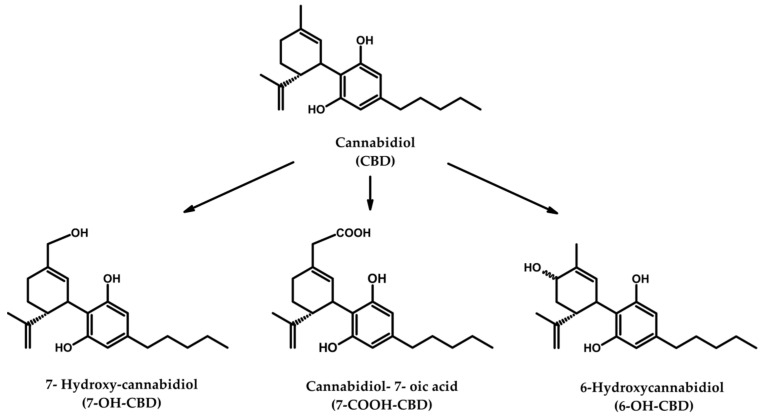
Chemical structure of CBD and its major metabolites.

Although UHPLC–MS/MS methods to analyze CBD or its metabolites were developed by our group [9,23], there is no analytical assay available for the simultaneous determination of CBD and its main metabolites (see Figure 1) in serum samples of individuals treated with CBD-based pharmaceuticals or medical cannabis. Therefore, we herein present a validated method discussed in the sections above. 

### 2.1. Validation of an Analytical Method

The method was tested over five succeeding days in serum samples following the criteria for bioanalytical method development and validation [24,25,26]. Selectivity, linearity, sensitivity (limits of detection (LOD) and quantification (LOQ)), accuracy, precision and carryover were calculated applying five different replicates of calibrators (six for each calibration curve) for five consecutive days and five replicates for three QC samples. The obtained results are shown in Table 1 and Table 2.

#### 2.1.1. Selectivity and Carry Over

**Figure 2:**
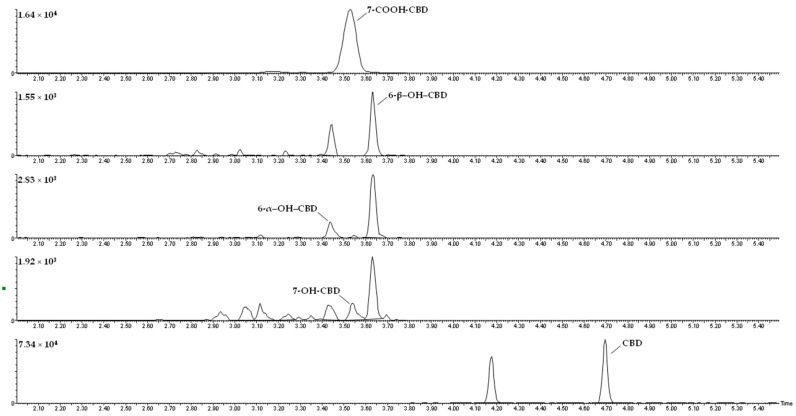
Chromatograms of a serum sample spiked with the analytes at the lower limit of quantification (LLOQ).

The chromatograms obtained for a serum sample spiked with the analytes at the lower limit of quantification (LLOQ) are presented in Figure 2. No interferences were detected at the retention times of the analytes. There was no signal of carryover when injecting the highest calibrator of the calibration curve subsequently to an injection of a drug-free serum sample.

#### 2.1.2. Linearity and Sensitivity

Previous studies have demonstrated that concentrations found for 6-α–OH–CBD and 6-β–OH–CBD were always lower than the ones for the major CBD metabolites [4]. For this reason and the study of linearity, six calibrators were selected in the range of 0.5–25 ng/mL for 6-α–OH–CBD and 6-β–OH–CBD, 1–500 for CBD and 7-OH-CBD and 20–10,000 for 7-COOH-CBD. Quality controls (QC) solutions were prepared at the concentrations of 0.75 ng/mL (low QC), 3 ng/mL (medium QC) and 20 ng/mL (high QC) for 6-α–OH–CBD and 6-β–OH–CBD; and 0.5 ng/mL (low QC), 50 ng/mL (medium QC) and 333 ng/mL (high QC) for the remain analytes. Linearity was evaluated every day of validation with determination coefficients (r^2^) equal to or higher than 0.995. Limits of quantification (LOQ) obtained for all the analytes fitted for the study. According to Peters et al., accuracy and precision were within ±20% at LOQ for all matrices and within ±15% at all the QC samples [24]. The obtained LLOQ was considered the lowest concentration measured with a coefficient of variation (CV) ≤ 20% and a relative error (RE) within ±20%.

#### 2.1.3. Precision and Accuracy

The valuation of inter-day precision and accuracy was made throughout 5 days with a six concentration levels. The coefficients of variation (CVs) were typically lower than 15% for all analytes at the concentration levels within an ±20% inaccuracy interval. To study the intra-day precision, six replicates of blank serum samples spiked with the target analytes at three concentration levels (low QC, 0.75 ng/mL; medium QC, 3 ng/mL; high QC, 20 ng/mL for 6-α–OH–CBD and 6-β–OH–CBD; low QC; 0.5 ng/mL; medium QC, 50 ng/mL and high QC, 333 ng/mL for CBD, 7-OH-CBD and 7-COOH-CBD) were analyzed in the same day. Results showed a CV lower than 20% with a mean relative error (RE) within 15% for the tested concentrations.

#### 2.1.4. Recovery and Matrix Effect

Three different concentrations were tested, the lower QC (0.5 ng/mL; 0.75 ng/mL), the higher QC (333 ng/mL; 20 ng/mL) and an intermediate QC (50 ng/mL; 3 ng/mL). The method showed recoveries ranging from 90.1–100.4% for CBD and metabolites. As for matrix effect, results showed a range from 52.7% to 109.2%.

#### 2.1.5. Analysis of Patients’ Samples

The novel method was applied on twelve samples derived from patients under treatment with different CBD formulations and dosages. It is relevant to point out that some patients gave more than one sample for further analysis (patients 3 to 5).

**Figure 3:**
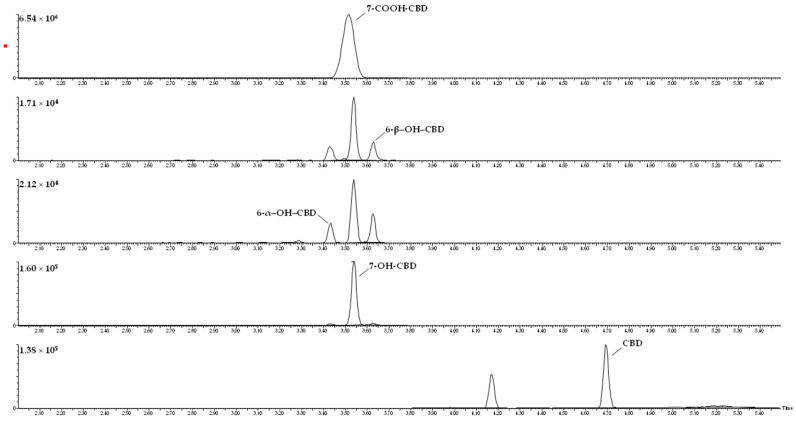
Chromatograms for each analyte from a real serum sample.

Serum levels of CBD (88.20–396.31 and 13.19–170.63 ng/mL) as well as of 7-OH-CBD (27.11–313.63 and 14.01–77.52 ng/mL) and 7-COOH-CBD (380.32–10112.23 and 300.57–2851.82 ng/mL) (shown in Table 2) were significantly higher (*p* < 0.05) in patients treated with GW pharma CBD compared to those of patients treated with galenic preparations. 6-α–OH–CBD and 6-β–OH–CBD were detected in the first group (patient 1 to 5) and were undetectable in the second group (patients 6 to 9). 7-COOH-CBD was confirmed as the most abundant metabolite in serum (5–10 fold higher than CBD) followed by 7-OH CBD. A significant correlation between dose administered and CBD concentration (*p* < 0.05) and a higher bioavailability were confirmed in patients treated with GW pharma CBD preparation (see Table 3). Figure 3 presents the chromatograms for each analyte after the analysis of a real sample.

#### 2.1.6. Sample Stability

As previously established [9] some degradation was observed after three freeze/thaw cycles, with concentrations within 10% of target for all the compounds under investigation. Similar results were obtained in serum QC samples analyzed before and after hydrolysis process. In addition, five aliquots for each QC sample were re-analized after three months storage at −20°C and no relevant degradation was observed. A limitation found in this study was that the stability of the analytes in the presence of other co-medications administered to patients involved in the study was not assessed, since each individual took different medications, not known at the moment of analytical method development.

## 3. Materials and Methods

### 3.1. Chemicals and Reagents

Working standards of CBD and CBD metabolites, i.e., 7-COOH-CBD, 7-OH-CBD, 6-α–OH–CBD and 6-β–OH–CBD were purchased from Dalton Research Molecules (Toronto, ON, Canada) and deuterated internal standards (ISs), i.e., CBD-d_3_ and 11-hydroxy-THC-d_3_ (11-OH-THC-d_3_), were purchased from Cayman Chemical (Ann Arbor, MI, USA) and stored at −20 °C until use. LC-MS grade water, acetonitrile and formic acid and LC grade acetone were obtained from Sigma-Aldrich^®^ (Milano, Italy). Ammonium formate 5 mM was prepared with 97% purity ammonium formate ammonium salt (Sigma-Aldrich^®^) dissolved in LC-MS grade water. M3^®^ reagent and precipitant solvent were acquired from Comedical^®^ s.r.l. (Trento, Italy).

### 3.2. Instrumental Conditions for UHPLC-MS/MS

UHPLC-MS/MS analysis was carried out on a Waters^®^ Xevo^®^ TQ-S micro mass spectrometer (triple quadrupole) prepared with an electrospray ionization source operating in negative-ion mode (ESI^−^) and interfaced with an ACQUITY UPLC^®^ I-Class (Waters^®^; Milano, Italy). Data was obtained with MassLynx^®^ software version 4.1 (Waters^®^, Milano, Italy). Separation was performed on an ACQUITY UPLC^®^ BEH C18 column from Waters^®^ (Milano, Italy) (length: 100 mm, internal diameter: 2.1 mm, particle size: 1.7 μm). Run time was 8 min with a gradient mobile phase composed by ammonium formate 5 mM at pH 7.5 (A) and acetonitrile (B) at a flow rate of 0.4 mL/min. Initial conditions were 5% B, held for 0.25 min, increased gradually to 100% B within 5.3 min, decreased to 5% B within 5.4 min, held for 2.6 min. Autosampler and column oven temperatures were 10 °C and 50 °C, respectively. The mass spectrometer operated in scheduled multiple reaction monitoring (MRM) mode, with two transitions for each analyte and IS (see Table 4). MS parameter settings were optimized by infusing neat standards individually in methanol and ramping cone voltage and collision energy (see Table 4). Scan speed (dwell time) was 0.023 s. ESI conditions were optimized as follows: capillary voltage = −2.8 kV, source temperature = 150 °C, desolvation temperature = 650 °C, cone gas flow rate = 0.18 mL/min, desolvation gas flow rate = 1200 L/h.

### 3.3. Preparation of Calibration Standards and Quality Control Samples

Standard stock solutions with all five non-deuterated standards were prepared in methanol at 1 μg/mL, 10 μg/mL, 100 μg/mL and 1 mg/mL. Internal standard (IS) stock solution with 11-OH-THC-d_3_ was prepared in methanol at 1 µg/mL. Deuterated standard of 11-OH-THC was used due to the inaccessibility of deuterated standards of CBD metabolites at the time of the analysis. Stock solutions were stored in glass vials at −20 °C. Calibrator working solutions were prepared extemporaneously in methanol from the standard stock solutions (6 calibrators at 0.5, 1, 5, 15 and 25 ng/mL for 6-α–OH–CBD and 6-β–OH–CBD; 6 calibrators at 5, 10, 50, 100 and 200 ng/mL for CBD and 7-OH-CBD; 6 calibrators at 0, 250, 500, 2000, 5000 and 10,000 ng/mL for 7-CBD-COOH). Low, medium and high-quality control (QC) working solutions were daily prepared from the standard stock solutions in methanol. They contained all analytes at 0.75, 3 and 20 ng/mL for 6-α–OH–CBD and 6-β–OH–CBD; and 0.5, 50, and 333 ng/mL for the remaining compounds.

### 3.4. Sample Preparation

Serum samples were extracted after alkaline hydrolysis since previous studies and our preliminary experiments in real samples showed that CBD metabolites were all present as glucuronides serum samples [27]. Glucuronide hydrolysis was conducted in basic conditions, adding 5 μL IS solution (100 ng/mL), 10 μL of 10 M potassium hydroxide to 50 μL serum and heating at 100 °C for 30 min. After hydrolysis, 2.5 μL formic acid (≥99.9%) was added and 50 μL hydrolyzed samples were collected into a polypropylene microcentrifuge tube (Safe-Lock Tube^®^, Eppendorf, Milano, Italy). These samples were added with 50 μL M3^®^ buffer reagent, to preserve the stability of the analyte, and 200 μL acetone: acetonitrile (8:2, *v*/*v*) in polypropylene microcentrifuge tubes. Tubes were then capped, vortexed for 10 s and centrifuged at 5000× *g* for 5 min. Supernatants (200 μL) were transferred into autosampler glass vials, before injection of 10 μL onto the chromatographic system.

### 3.5. Validation of the Analytical Method

#### 3.5.1. Selectivity, Sensitivity and Linearity

This parameter measured the capability to identify the analytes under study in the presence of matrix components. Blank serum samples were checked for endogenous interferences. In addition, the method’s specificity was also studied. Serum samples were checked for eventual interferences from other drugs.

The calibration curves resulted in the peak area ratio between each compound and the corresponding IS versus the correspondent concentration.

#### 3.5.2. Precision and Accuracy

The study of inter-day precision and accuracy was performed during five days with 6 concentration levels. The precision, expressed as the RSD (%), and the accuracy was calculated as (determined/nominal concentration) × 100%. The acceptance criterion for the precision and accuracy was set to a CV < 20% with a relative error within 15%.

#### 3.5.3. Recovery and Matrix Effect

The relative peak areas obtained from the extracted compounds (adding the compounds before extraction) were compared to relative peak areas obtained from samples that were spiked with the compounds after extraction (100% recovery). The ISs mixture was added to both sets after extraction.

### 3.6. Application on Patients Samples

The suitability of the developed method on real samples was tested on clinical samples derived from nine patients under treatment with different formulations of CBD (five with GW pharma CBD and four with galenic preparations as CBD extract oils and crystals) for the treatment of drug-resistant epilepsy. Patients were children and young adults followed up at the Giannina Gaslini Children’s Hospital: six males (age: 3–26 years; weight: 16–80 kg) and four females (age 6–11 years; weight 22–45 kg). The study was approved by the Regional Ethical Committee (CER Liguria: 056/057/058/059-2019) and written informed consent was signed by patients or caregivers. Table 5 summarizes the patients’ demographics.

## 4. Conclusions

TDM is of strong support in the dose-adjustment and clinical management of patients taking different anti-seizure medications (ASMs) (e.g., valproate, carbamazepine, or CBD). Though the preferred collection method is that of venous blood, yet some new easily and patients-friendly methods such that of peripheral capillary microsampling have been recently developed and effectively applied in the clinical practice for the TDM of CBD [17,18]. Beyond this, one of the current limitations of CBD-based treatments is that both purified and galenic preparations are available yielding high interindividual variability and even some limits in the TDM. The fast and simple UHPLC-MS/MS method developed in this study enabled the robust and sensitive quantification of CBD it metabolites and has proven to be precise, accurate and highly efficient by avoiding the serum matrix effect and enabling the reproducible recovery of the analytes thought an alkaline hydrolysis reaction. The method was applied to clinical samples derived from nine patients under treatment with different formulations of CBD for the treatment of drug-resistant epilepsy. Significantly higher and more stable serum levels of either CBD or its metabolites were detected in those patients taking the purified formulation of CBD as compared to those treated with galenic preparations. From a clinical perspective, these findings may suggest patients treated with GW pharma CBD formula have more drug “coverage” between the daily dose intakes, translating into better seizure control. 

## Figures and Tables

**Table 1:** Linear range, linear equation, determination coefficient, limit of detection (LOD) and limit of quantification (LOQ) for the analytes in serum samples.

Compounds	Linear Range (ng/mL)	Linear Equation	Determination Coefficient (r^2^)	LOD (ng/mL)	LOQ (ng/mL)
CBD	0–500	*y* = 0.002*x* + 0.001	0.997 ± 0.002	0.17	1.0
7-COOH-CBD	0–10,000	*y* = 0.034*x* + 0.020	0.998 ± 0.001	0.72	20.0
7-OH-CBD	0–500	*y* = −7.119*x* + 0.009	0.999 ± 0.001	0.11	1.0
6-α–OH–CBD	0–25	*y* = 0.147*x* + 0.006	0.995 ± 0.005	0.04	0.5
6-β–OH–CBD	0–25	*y* = 0.047*x* + 0.014	0.999 ± 0.013	0.02	0.5

Abbreviations: CBD, cannabidiol; 7-COOH-CBD, cannabidiol-7-oic acid; 7-OH-CBD, 7-hydroxycannabidiol; 6-α–OH–CBD, 6-α-hydroxycannabidiol; 6-β–OH–CBD, 6-β-hydroxycannabidiol; LOD, limit of detection; LOQ, limit of quantification.

**Table 2:** Validation parameters for the analytes in serum samples.

Compounds	QC Concentrations (ng/mL)			Accuracy (%)			Intra-Assay Precision (%CV)			Inter-Assay Precision (%CV)			Recovery (%)			Matrix Effect (%)		
	**Low QC**	**Medium QC**	**High QC**	**LowQC**	**Medium QC**	**HighQC**	**LowQC**	**Medium QC**	**HighQC**	**LowQC**	**Medium QC**	**HighQC**	**LowQC**	**Medium QC**	**High QC**	**Low QC**	**Medium QC**	**High QC**
CBD	0.5	50.0	330.0	10.7	8.4	6.2	14.2	9.4	1.0	13.9	10.2	6.9	90.1	92	95.8	52.7	62.5	53.7
7-COOH-CBD	0.5	50.0	330.0	6.5	8.7	5.8	7.2	0.4	4.1	9.1	7.4	5.7	90.6	93.9	96.1	109.2	98.2	98.4
7-OH-CBD	0.5	50.0	330.0	5.5	9.7	4.3	4.2	4.2	3.7	6.8	3.1	2.8	94.9	97.4	98.3	95.5	98.4	92.4
6-α–OH–CBD	0.75	3.0	20.0	8.5	6.2	7.9	10	2.1	4.4	6.7	4.5	8.3	91.9	98.6	96.9	84.4	85.7	95.9
6-β–OH–CBD	0.75	3.0	20.0	5.9	6.6	4.2	8.9	8.1	6.4	8.2	6.1	5.8	94.3	97.5	100.4	86.5	94.6	102.3

Abbreviations: CBD, cannabidiol; 7-COOH-CBD, cannabidiol-7-oic acid; 7-OH-CBD, 7-hydroxycannabidiol; 6-α–OH–CBD, 6-α-hydroxycannabidiol; 6-β–OH–CBD, 6-β-hydroxycannabidiol; QC, quality control; CV, coefficient of variation.

**Table 3:** Formulation, doses and concentration detected for CBD and metabolites in patients.

Patient ID	Formulation	Dose of CBD (mg/kg/die)	Concentration (ng/mL)				
			6-α-OH-CBD	6-β-OH-CBD	7-OH-CBD	CBD-COOH	CBD
1	GW pharma CBD	15.25	1.15	0	27.11	380.32	239.74
2	GW pharma CBD	17.00	12.46	7.60	313.63	9707.01	279.75
3	GW pharma CBD	9.25	4.97	2.17	298.16	10,112.23	130.12
		8.15	4.02	1.33	286.99	8849.05	105.74
4	GW pharma CBD	20.00	9.04	10.14	169.39	1510.89	343.81
			24.45	19.13	272.55	3200.88	396.31
5	GW pharma CBD	17.20	0	0.76	115.48	3030.12	80.29
			4.03	4.48	205.36	6616.54	170.63
6	BEDROLITE + BEDICA + pure CBD	1.20	0	0	14.01	300.57	13.19
7	BEDROLITE	6.70	0	0	42.34	2625.34	23.33
8	ENECTA CBD Oil	4.22	0	0	48.73	609.89	36.02
9	BEDROLITE + CBD crystal	27.00 (1 + 26)	0	0	77.52	2851.82	36.58

Abbreviations: 6-α–OH–CBD, 6-α-hydroxycannabidiol; 6-β–OH–CBD, 6-β-hydroxycannabidiol; 7-OH-CBD, 7-hydroxycannabidiol; 7-COOH-CBD, cannabidiol-7-oic acid; CBD, cannabidiol.

**Table 4:** Mass spectrometry parameters for analytes and internal standards.

Compounds	Internal Standard	Cone Voltage (v)	Q1 Mass (m/z)	Quantification Transition		Confirmation Transition		RT (min)
				Q3 Mass (m/z)	CE (eV)	Q3 Mass (m/z)	CE (eV)	
6-α–OH–CBD	11-OH-THC-d_3_	30	329.2	158.2	32	173.1	28	3.43
7-OH-CBD	11-OH-THC-d_3_	40	329.1	261.2	20	268.1	24	3.53
7-COOH-CBD	11-OH-THC-d_3_	40	343.1	179.2	20	231.2	26	3.54
6-β–OH–CBD	11-OH-THC-d_3_	30	329.2	158.2	30	173.2	30	3.62
CBD	CBD-d_3_	40	313.3	107.1	40	245.2	40	4.69
11-OH-THC-d_3_	-	30	332.2	173.1	30	271.1	30	4.16
CBD-d_3_	-	45	316.1	110.1	45	248.2	45	4.69

Abbreviations: 6-α–OH–CBD, 6-α-hydroxycannabidiol; 7-OH-CBD, 7-hydroxycannabidiol; 7-COOH-CBD, cannabidiol-7-oic acid; 6-β–OH–CBD, 6-β-hydroxycannabidiol; CBD, cannabidiol; 11-OH-THC, 11-hydroxy-Δ^9^-tetrahydrocannabinol; CE, collision energy; RT, retention time.

**Table 5:** Patients’demographics.

Patient ID	Age, Gender	Weight (kg)	Disorder
1	17, *♂*	42.0	Dravet syndrome
2	26, *♂*	80.0	Dravet syndrome
3	12, ♀	76.0	Dravet syndrome
4	6, ♀	22.5	Dravet syndrome
5	8, *♂*	23.3	Dravet syndrome
6	3, *♂*	16.7	Drug-resistant epilepsy
7	5, *♂*	26.8	Drug-resistant epilepsy
8	11, ♀	43.1	Rett syndrome
9	11, *♂*	28.4	Drug-resistant epilepsy

## Data Availability

The data presented in this study are available on demand.
